# Acute stress and multicellular development alter the solubility of the *Dictyostelium* Sup35 ortholog ERF3

**DOI:** 10.1128/spectrum.01607-24

**Published:** 2024-09-30

**Authors:** Felicia N. Williams, Kanesha L. Travis, Holly N. Haver, Anna D. Umano, Yaneli Guerra-Hernandez, K. Matthew Scaglione

**Affiliations:** 1 Department of Molecular Genetics and Microbiology, Duke University, Durham, North Carolina, USA; 2 Center for Neurodegeneration and Neurotherapeutics, Duke University, Durham, North Carolina, USA; University of Mississippi, University, Mississippi, USA

**Keywords:** *Dictyostelium*, prion, protein aggregation, multicellular development, cellular stress

## Abstract

Among sequenced organisms, the genome of *Dictyostelium discoideum* is unique in that it encodes for a massive amount of repeat-rich sequences in the coding region of genes. This results in the *Dictyostelium* proteome encoding for thousands of repeat-rich proteins, with nearly 24% of the *Dictyostelium* proteome encoding Q/N-rich regions that are predicted to be prion like in nature. To begin investigating the role of prion-like proteins in *Dictyostelium*, we decided to investigate ERF3, the *Dictyostelium* ortholog of the well-characterized yeast prion protein Sup35. ERF3 lacks the Q/N-rich region required for prion formation in yeast, raising the question of whether this protein aggregates and has prion-like properties in *Dictyostelium*. Here, we found that ERF3 formed aggregates in response to acute cellular stress. However, unlike *bona fide* prions, we were unable to detect transmission of aggregates to progeny. We further found that aggregation of this protein is driven by the ordered C-terminal domain independently of the disordered N-terminal domain. Finally, we also observed aggregation of ERF3 under conditions that induce multicellular development, suggesting that this phenomenon may play a role in *Dictyostelium* development. Together, these findings suggest a role for regulated protein aggregation in *Dictyostelium* cells under stress and during development.

**IMPORTANCE:**

Prion-like proteins have both beneficial and deleterious effects on cellular health, and many organisms have evolved distinct mechanisms to regulate the behaviors of these proteins. The social amoeba *Dictyostelium discoideum* contains the highest proportion of proteins predicted to be prion like and has mechanisms to suppress their aggregation. However, the potential roles and regulation of these proteins remain largely unknown. Here, we demonstrate that aggregation of the *Dictyostelium* translation termination factor ERF3 is induced by both acute cellular stress and by multicellular development. These findings imply that protein aggregation may have a regulated and functional role in the *Dictyostelium* stress response and during multicellular development.

## INTRODUCTION

Under conditions of cellular stress, proteins can come together to form a wide variety of higher order structures, ranging from small oligomeric assemblies to large protein aggregates. While these various assemblies can adversely affect the cell, there can also be functional benefits to protein assembly under stress (1–6). Prions are one class of proteins where these beneficial and adverse cellular impacts have been widely observed. In yeast, aberrant aggregation of prion proteins can lead to several deleterious effects, including growth defects, formation of insoluble protein aggregates, and interference with normal cellular processes. However, aggregation of prion proteins can also assist cells in surviving acute stress, aid in recovery from dormancy, and ameliorate polyglutamine toxicity (1–13).

One protein where this dichotomy has been well illustrated is the *Saccharomyces cerevisiae* prion-forming protein Sup35. Sup35 forms the [*PSI+*] prion in *S. cerevisiae* and is an essential eukaryotic release factor necessary for translation termination (14–17). In cells, prion formation by Sup35 occurs in response to acute stress, alters the aggregation of polyglutamine proteins, and impacts overall cellular fitness (5, 12, 13, 18–28). The protein consists of three functional domains: a Q/N-rich N-terminal domain, a charged middle domain, and a C-terminal GTPase domain (18, 29, 30). The Q/N-rich N-terminal domain is required for [*PSI+*] formation in *S. cerevisiae*, and the composition of this domain is an important determinant of prion formation across various fungal species (29–33). The middle domain is rich in negatively charged residues and can impact protein solubility (12, 34, 35). Together, the disordered NM domains can drive amyloidogenic protein aggregation and have been widely studied as a model for this process (19, 32, 36, 37). The ordered C-terminus of Sup35 contains the GTPase domain responsible for the translational termination activity of the protein (30, 38). Notably, the C-terminal domain can aggregate independently of the NM domains, with some evidence suggesting that the NM domains assist in maintaining protein solubility despite their amyloid forming capability (12, 39, 40). This domain is also responsible for mediating the interaction with the translation termination cofactor Sup45 and is also sufficient for deposition of Sup35 into stress granules (39–41).

Outside of fungi, the biology of Sup35 and other microbial prion proteins is largely unknown. The proteome of *Dictyostelium discoideum* contains the highest proportion of proteins resembling known prions among sequenced organisms (42–45). Among these are thousands of proteins containing low-complexity domains and homopolymeric amino acid repeats (45–47). While these features generally promote aggregation, formation of insoluble protein aggregates is largely suppressed in *Dictyostelium* (45, 48, 49). For instance, proteins containing expanded polyglutamine tracts that would form toxic aggregates in most species remain soluble in *Dictyostelium* (45, 49). In addition, a particularly aggregation-prone variant of the Sup35 NM domains remains soluble in *Dictyostelium* in the absence of additional stressors but can aggregate with heat stress (45). Prion-like proteins in *Dictyostelium* are functionally enriched for specific protein types including kinases, transcription factors, and RNA-binding proteins (45, 46). Interestingly, prion-like proteins in *Dictyostelium* are also functionally enriched in processes related to multicellular development (44). Taken together, these findings suggest that there may be a functional role for the highly repetitive and prion-like proteins of the *Dictyostelium* proteome. Despite this, little investigation has been done to clarify the potential roles of these proteins in *Dictyostelium*.

Here, we sought to identify orthologs of well-studied yeast prion proteins in *Dictyostelium* and determine whether prion-like behaviors are conserved. To accomplish this, we investigated the eukaryotic release factor ERF3, the *Dictyostelium* ortholog of the prion-forming yeast protein Sup35. While *Dictyostelium* ERF3 shares several key features with yeast Sup35, ERF3 lacks the Q/N-rich domain necessary for prion formation in yeast. Notably, we found that ERF3 forms higher molecular weight aggregates in response to acute stress; however, these aggregates did not persist in a prion-like manner. We also found that ERF3 aggregation occurred independently of the disordered N-terminal domain and instead was driven by the structured C-terminal domain. Strikingly, we also found that ERF3 aggregation occurs under nutrient stress and during multicellular development. Together, these findings suggest a potential role for regulated protein aggregation in the *Dictyostelium* stress response and during multicellular development.

## RESULTS

### Cellular stress induces ERF3 protein aggregation in *Dictyostelium*

**Fig 1:**
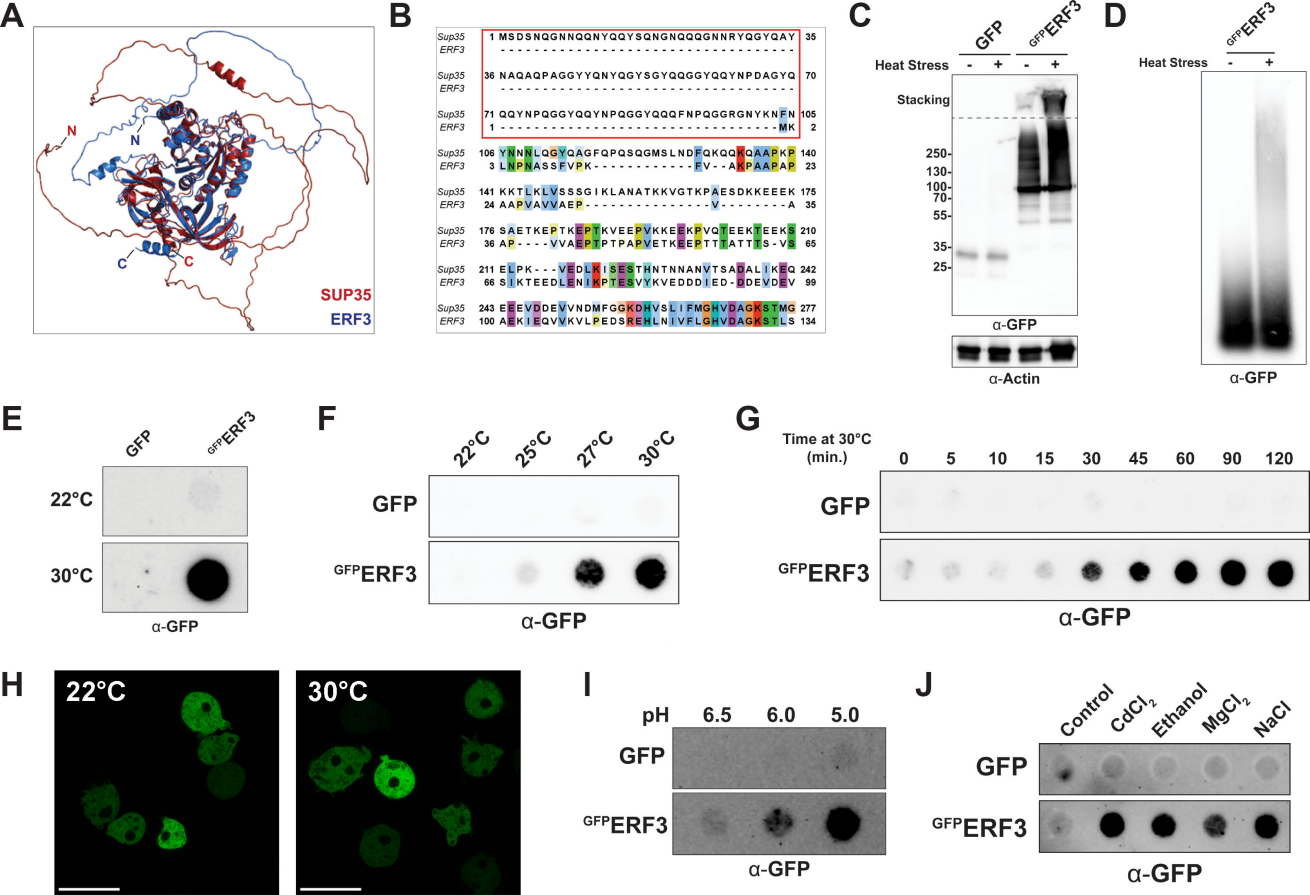
ERF3 forms SDS-insoluble aggregates in response to cellular stress. (**A**) *Dictyostelium* ERF3 and *S. cerevisiae* Sup35 are structurally similar. Protein structures predicted by AlphaFold and aligned in PyMOL. (**B**) ERF3 lacks the Q/N-rich prion domain of yeast Sup35. Protein sequences aligned by ClustalOmega and visualized in Jalview. The Sup35 prion domain (residues 1–103) is outlined in red. (**C–E**) ERF3 forms SDS-resistant aggregates during heat stress. Lysates from transiently heat stressed *Dictyostelium* cells expressing either GFP or ^GFP^ERF3 were analyzed by SDS-PAGE (**C**), semi-denaturing detergent agarose gel electrophoresis (SDD-AGE) (**D**), and filter trap (**E**) followed by western blot (*n* = 3). (**F**) ^GFP^ERF3 aggregates at moderate heat stress temperatures. Cells were transiently heat stressed at the indicated temperatures and analyzed by filter trap and western blot (*n* = 3). (**G**) ^GFP^ERF3 aggregates within 30 minutes of heat stress. Cells were subjected to heat stress for the indicated times and then analyzed by filter trap and western blot (*n* = 3). (**H**) Insoluble ^GFP^ERF3 aggregates are not visible by super-resolution confocal microscopy. *Dictyostelium* cells expressing ^GFP^ERF3 were incubated at the indicated temperatures and then imaged by confocal microscope with Airyscan super-resolution. Scale bars represent 20 µm. (**I**) Growth in acidic media induces ^GFP^ERF3 aggregation. Cells were grown in HL5 media at a standard pH of 6.5 or at an acidic pH of 6.0 or 5.0 and then analyzed by filter trap and western blot (*n* = 3). (**J**) Other cellular stressors also induce ^GFP^ERF3 aggregation. Cells were incubated with empty control, oxidative stress (200 µM CdCl_2_ or 2% ethanol), chaotropic stress (100 mM MgCl_2_), or osmotic stress (0.15 M NaCl) and then analyzed by filter trap and western blot (*n* = 3).

Because microbial prions have been studied most extensively in yeast, studies in yeast models have primarily guided the definition, characterization, and prediction of prion-like proteins across eukaryotes (14, 42, 47, 50–53). Thus, to begin our investigation into prion-like proteins in *Dictyostelium*, we sought to identify orthologs of well-defined yeast prion proteins. Using BLAST, we identified the translation termination factor ERF3 as the *Dictyostelium* ortholog of the yeast protein Sup35, which forms the [*PSI+*] prion. *Dictyostelium* ERF3 and *S. cerevisiae* Sup35 share strong sequence and structural homology in the C-terminal domain, as shown by an overlap in predicted structures (Fig. 1A). Other shared key characteristics include a disordered N-terminal domain and negatively charged middle domain (Fig. S1A and B). However, ERF3 lacks the Q/N-rich N-terminal domain necessary for [*PSI+*] prion formation in yeast (Fig. 1B). Based on this, we wanted to determine whether ERF3 retains prion-like characteristics in *Dictyostelium* despite lacking the Q/N-rich N-terminal domain.

We first wanted to determine whether the Q/N-rich N-terminal domain was dispensable for heat stress-induced aggregation of ERF3 in *Dictyostelium* as it is for Sup35 in yeast (12, 39). Although we attempted to monitor endogenous ERF3 solubility, there is not an existing antibody against *Dictyostelium* ERF3, and most commonly used antibodies against ERF3 orthologs were raised using non-homologous epitopes. While we attempted to use two antibodies raised against partially homologous regions of the human ERF3 ortholog, they failed to recognize *Dictyostelium* ERF3 (Fig. S2). Therefore, in order to detect changes in ERF3 solubility, we transiently heat stressed *Dictyostelium* cells overexpressing GFP-tagged ERF3 (^GFP^ERF3) and then assessed protein solubility by SDS-PAGE. Following heat stress, we observed signal in the stacking gel for ^GFP^ERF3 but not for GFP alone, consistent with the formation of ^GFP^ERF3 aggregates during heat stress (Fig. 1C). To better resolve these species, we used semi-denaturing detergent agarose gel electrophoresis (SDD-AGE) and again observed that a portion of ^GFP^ERF3 forms higher molecular weight assemblies in heat-stressed cells (Fig. 1D). Appearance of α-GFP signal by filter trap assay was also consistent with the formation of insoluble aggregates by ^GFP^ERF3 following heat stress (Fig. 1E). To evaluate how rapid and sensitive this response was, we next performed kinetic analyses to determine the time required for ^GFP^ERF3 aggregate formation and to assess aggregation at temperatures ranging between 22°C and 30°C. From these analyses, we observed that ^GFP^ERF3 aggregates form within 30 minutes of heat stress and at slightly elevated temperatures (27°C), suggesting that induction of ERF3 aggregation is a rapid and sensitive response to heat stress (Fig. 1F and G).

We next wanted to determine if these aggregates formed visible puncta within cells. To accomplish this, we imaged *Dictyostelium* cells expressing ^GFP^ERF3 with and without heat stress. Interestingly, despite ^GFP^ERF3 forming aggregates that could be detected biochemically, heat stress-induced ERF3 aggregates were not visible by super-resolution confocal microscopy (Fig. 1H). This suggests that these aggregates are morphologically distinct from the large puncta observed upon Sup35 aggregation in yeast and also from the Sup35-NM foci that can form in *Dictyostelium* under heat stress (12, 39, 40, 45, 54). Finally, to determine if ^GFP^ERF3 aggregation was induced upon other types of cellular stress, we treated *Dictyostelium* cells expressing ^GFP^ERF3 to a panel of cellular stressors. Consistent with ^GFP^ERF3 aggregation being a general stress response, we observed ^GFP^ERF3 aggregation in response to acidic stress by lowered pH of the media, oxidative stress by CdCl_2_ or ethanol, chaotropic stress by MgCl_2_, and osmotic stress by NaCl (Fig. 1I and J). Together, these findings suggest that despite lacking the Q/N-rich N-terminal domain, ERF3 does aggregate in response to cellular stress.

### ERF3 aggregates under stress, but these aggregates do not persist

**Fig 2:**
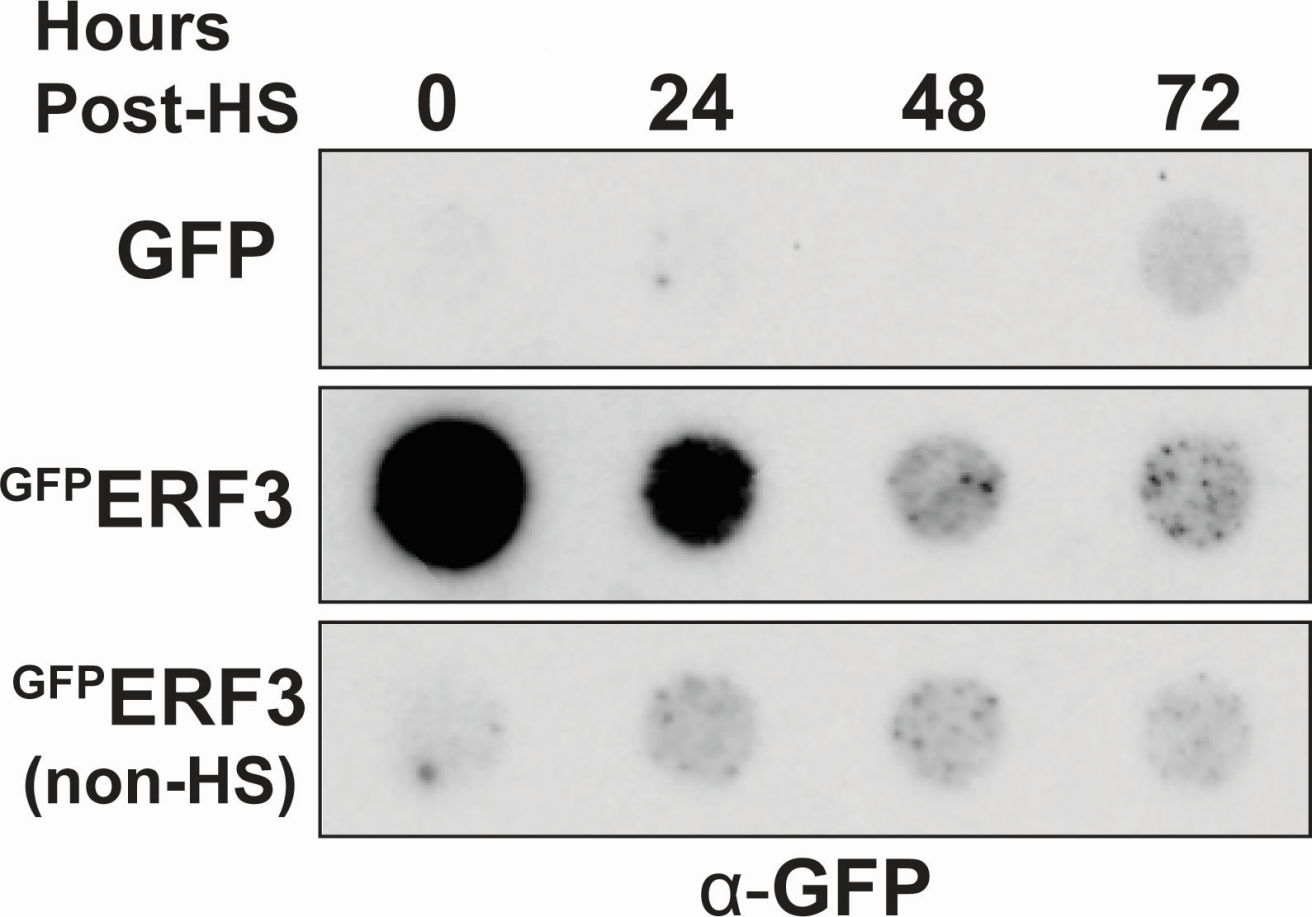
Prion-like persistence of heat stress-induced ^GFP^ERF3 aggregates was not detected. *Dictyostelium* cells expressing either GFP or ^GFP^ERF3 were transiently heat stressed and then passaged at permissive temperature. Lysates were collected daily and then analyzed by filter trap and western blot (*n* = 4).

The Q/N-rich N-terminal domain of Sup35 plays a critical role in driving its aggregation and is essential for prion formation and persistence (15, 37, 55, 56). Because ERF3 lacks this Q/N-rich domain, we wanted to determine if ^GFP^ERF3 aggregates persist upon the removal of stress. To accomplish this, we subjected *Dictyostelium* cells to transient heat stress before passaging the cells at permissive temperature. We then collected samples 24, 48, and 72 hours post-heat stress and performed filter trap analysis to determine if ^GFP^ERF3 aggregates persisted over time. Consistent with the Q/N-rich domain being necessary for persistence, heat stress-induced ^GFP^ERF3 aggregates were no longer visible within 48 hours (Fig. 2). This suggests that stress-induced ^GFP^ERF3 aggregates do not persist in a prion-like manner. However, given that prion conversion is a rare event in yeast, we cannot rule out the possibility that rare instances of persistence occur in *Dictyostelium* but are below the level of detection in our assay.

### ERF3 self-assembles *in vitro*

**Fig 3:**
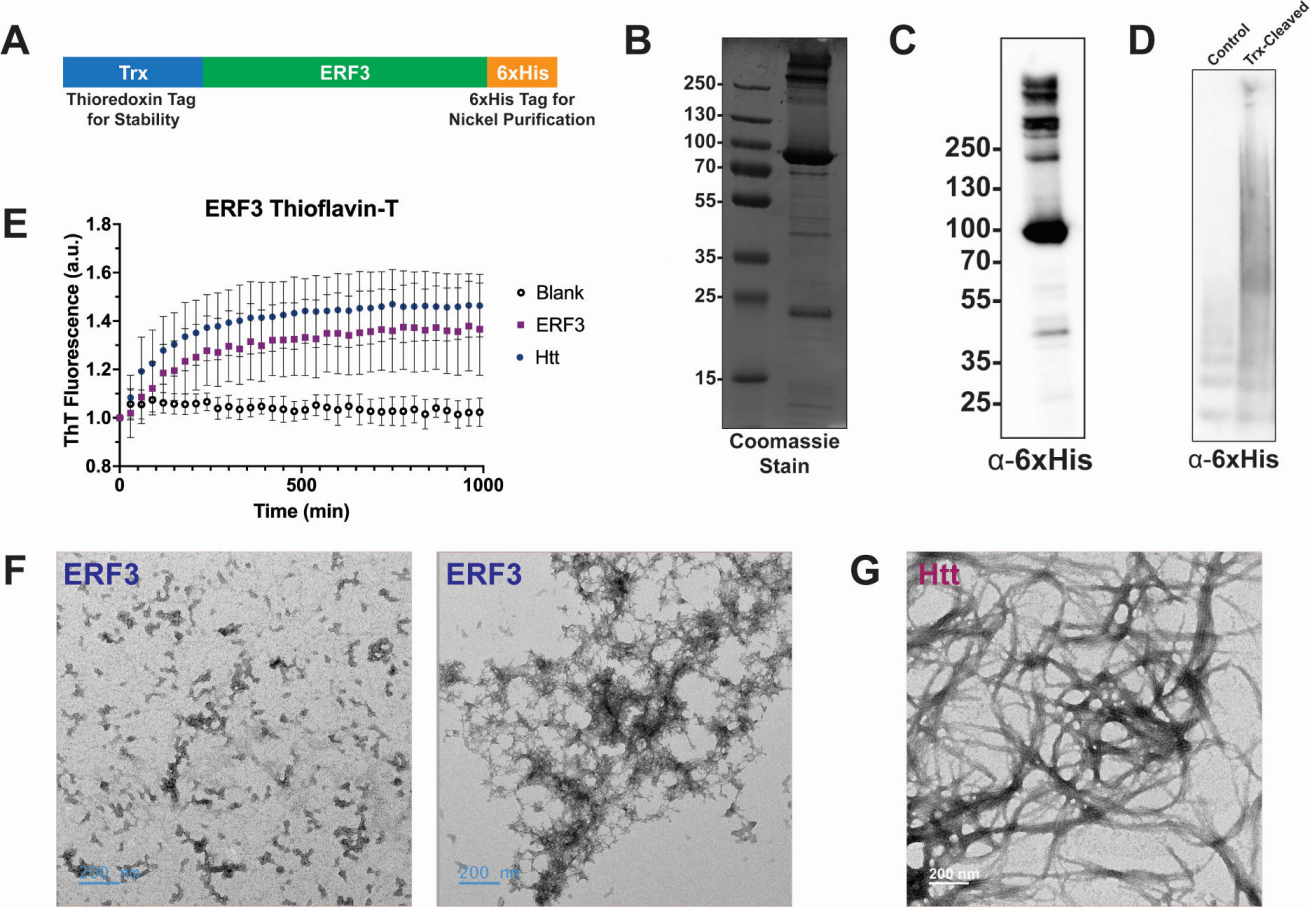
ERF3 self-assembles *in vitro*. (**A**) Schematic of the recombinant ERF3 construct. The recombinant ERF3 contains a cleavable Trx tag on the N-terminus to stabilize the protein and a 6xHis tag on the C-terminus to enable nickel purification. (**B and C**) The purified ^Trx^ERF3 protein runs at its expected size (77 kDa). SDS-PAGE gels of the recombinant ^Trx^ERF3 protein were either Coomassie stained (**B**) or analyzed by western blot (**C**). Purified ^Trx^ERF3 protein runs at its expected size of 77 kDa and also ladders at higher molecular weights, indicating potential oligomer formation. (**D**) Removal of the Trx tag results in ERF3 aggregation. Recombinant ^Trx^ERF3 was incubated with empty vehicle or with enterokinase and then analyzed by SDD-AGE and western blot (*n* = 5). (**E**) Trx-cleaved ERF3 produces signal by ThT. Recombinant ^Trx^ERF3 or Htt^ex1Q46^ were incubated with enterokinase, and fluorescence of the amyloid-specific dye ThT was monitored (*n* = 3; error bars indicate standard deviation). (**F and G**) TEM micrographs of Trx-cleaved recombinant ERF3 and mutant Htt protein. Trx-cleaved ERF3 (**F**) forms large, branched assemblies while Trx-cleaved Htt (**G**) produces highly ordered fibrils.

Protein aggregation can be intrinsically driven or induced by the presence of other protein aggregates (7, 13, 20, 24–28). Therefore, we wanted to establish an *in vitro* system to determine if ERF3 alone was sufficient to form aggregates independently of the Q/N-rich *Dictyostelium* proteome. To do this, we developed a construct for bacterial expression of ERF3 containing a C-terminal 6xHis tag to enable purification and an N-terminal thioredoxin (Trx) tag to stabilize the protein (^Trx^ERF3; Fig. 3A). Interestingly, besides running at the expected molecular weight of 77 kDa, the purified ERF3 also formed a laddering pattern at higher molecular weights suggesting that the protein may be forming small oligomeric species *in vitro* (Fig. 3B and C). To determine if recombinant ERF3 can self-assemble into large aggregates *in vitro*, we cleaved off the stabilizing Trx tag and assessed the formation of ERF3 aggregates by SDD-AGE. Trx-cleaved ERF3 formed higher molecular weight assemblies, consistent with self-assembly *in vitro* (Fig. 3D). We also monitored the kinetics of aggregation using the amyloid-specific dye thioflavin-T (ThT) and found that Trx-cleaved ERF3 produced ThT signal with similar kinetics to a mutant huntingtin (Htt) exon 1 positive control, suggesting that ERF3 may be forming amyloid *in vitro* (Fig. 3E). However, transmission electron microscopy (TEM) of the Trx-cleaved ERF3 displayed a mixture of small assemblies and large, branching assemblies distinct from the highly ordered fibrils produced by Trx-cleaved Htt (Fig. 3F and G). Together, the formation of high-molecular weight assemblies, production of ThT signal, and presence of branching structures observed by TEM are consistent with ERF3 self-assembling *in vitro* and suggests that ERF3 may form amyloid *in vitro*.

### The C-terminal domain drives ERF3 aggregation

**Fig 4:**
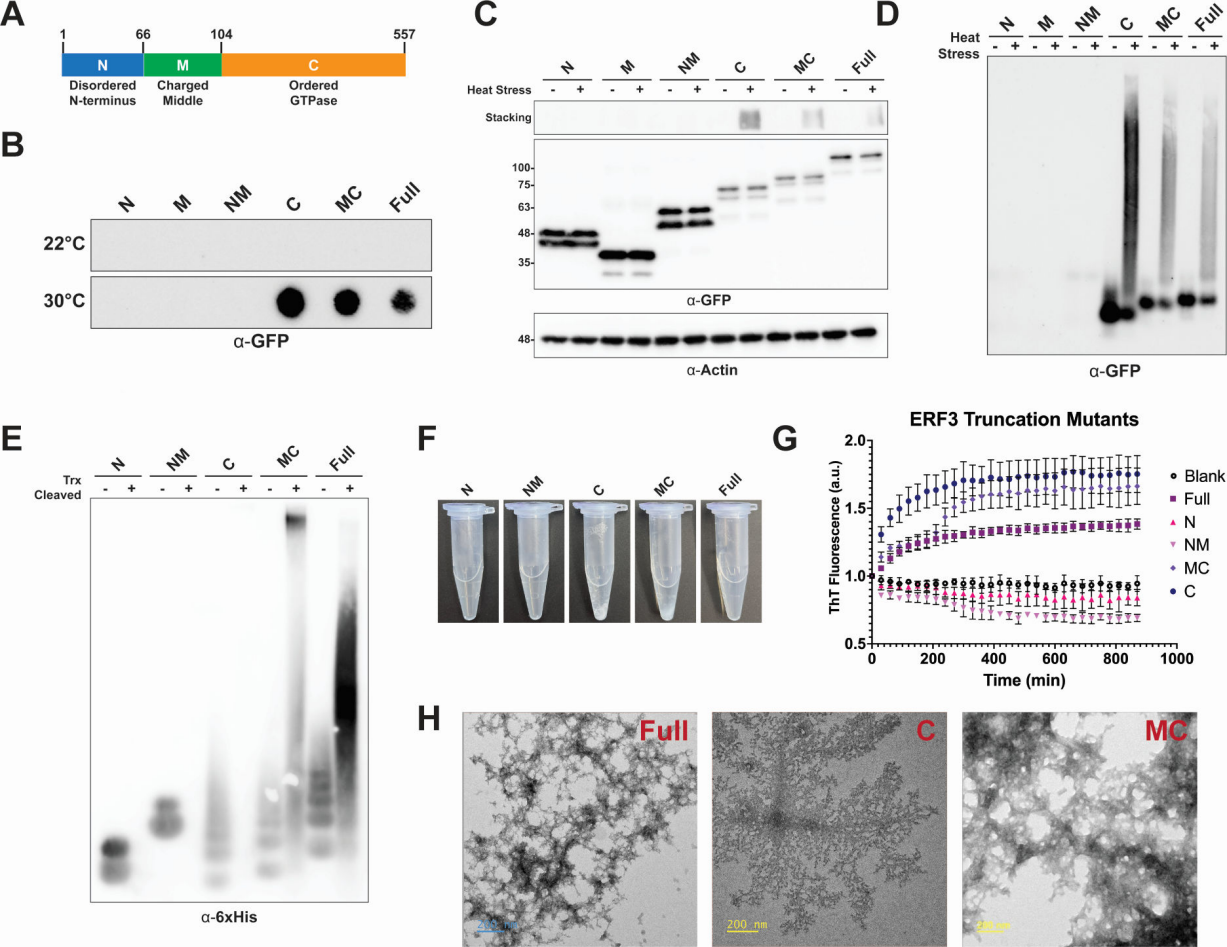
ERF3 aggregation is driven by the C-terminal domain. (**A**) Schematic representation of the domains of ERF3. Domains were estimated based on alignment with Sup35. (**B–D**) The C-terminal domain is required for ^GFP^ERF3 aggregation. *Dictyostelium* cells expressing GFP-tagged truncated or full-length ERF3 were heat stressed and then analyzed by filter trap assay (**B**), SDS-PAGE (**C**), and SDD-AGE (**D**) followed by western blot analysis (*n* = 3). (**E**) The C-terminal domain is required for aggregation of recombinant ERF3. Truncated or full-length ^Trx^ERF3 were incubated with enterokinase and then analyzed by SDD-AGE and western blot analysis (*n* = 2). (**F**) Trx-cleaved C and MC ERF3 truncations form visible white precipitate. Truncated or full-length ^Trx^ERF3 were incubated with enterokinase. Precipitated protein was pelleted by brief centrifugation; then, reaction tubes were photographed. (**G**) The C-terminal domain is required for potential amyloid formation. Truncated or full-length ^Trx^ERF3 were incubated with enterokinase, and ThT fluorescence was monitored (*n* = 3; error bars indicate standard deviation). (**H**) Recombinant ERF3 truncations containing the C-terminal domain form large assemblies. Truncated or full-length ^Trx^ERF3 were incubated with enterokinase and then visualized by TEM.

While aggregation of Sup35 in yeast can occur independently of the NM domains (12, 39, 40), formation of amyloid by yeast Sup35 has generally been associated with the Q/N-rich prion domain that is absent in *Dictyostelium* ERF3. Therefore, we next wanted to determine the domain requirements for ERF3 aggregation. To identify the domain that drives ERF3 aggregation, we generated GFP fusion constructs to express various combinations of the disordered N-terminal domain, the negatively charged middle domain, and the ordered C-terminal GTPase domains of ERF3 (Fig. 4A). We then overexpressed these constructs in *Dictyostelium* and subjected these cells to transient heat stress followed by SDS-PAGE, SDD-AGE, and filter trap analysis. From these analyses, we found that the C-terminal GTPase domain of ERF3 is necessary and sufficient to promote its aggregation in response to heat stress independently of the disordered N-terminal domain (Fig. 4B through D).

To confirm that this domain is also responsible for driving aggregation *in vitro*, we purified recombinant ERF3 domains and analyzed their assembly using SDD-AGE, ThT assay, and electron microscopy. In each instance, we found that the C-terminal domain was essential for ERF3 aggregation (Fig. 4E through H). By SDD-AGE, we saw that higher molecular weight assemblies only formed when the C-terminal domain was present (Fig. 4E). Notably, the C-terminal domain by itself did not produce signal (Fig. 4E); however, this is likely due to the rapid formation of large aggregates that are unable to be resolved by SDD-AGE as evidenced by the white precipitate that formed following Trx cleavage (Fig. 4F). The C-terminal domain was also required for production of ThT signal, with the C-terminal alone producing signal faster and to a higher degree than full-length ERF3 while the N and M domains did not produce ThT signal. Interestingly, the presence of the charged middle domain with the C-terminal domain resulted in biphasic aggregation kinetics, suggesting that the middle domain influences the aggregation of the C-terminal domain and may modify ERF3 aggregation (Fig. 4G). The three proteins containing the C-terminal domain also all produced branching structures observable by TEM. Together, these data identify the C-terminal domain of ERF3 as the key driver of ERF3 aggregation and possible amyloid formation.

### ERF3 aggregates during *Dictyostelium* development

**Fig 5:**
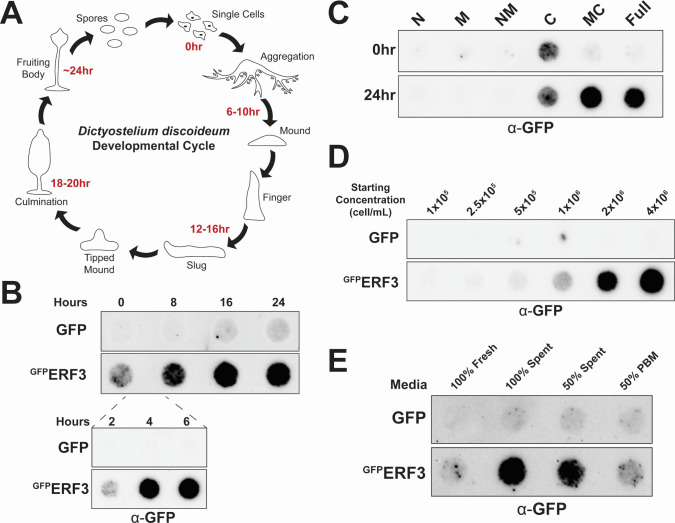
ERF3 aggregates during multicellular development. (**A**) Schematic representation of the *Dictyostelium* developmental cycle. *Dictyostelium* undergoes a developmental transition to multicellularity under starvation conditions. (**B**) ^GFP^ERF3 forms higher molecular weight assemblies during multicellular development. Development of *Dictyostelium* cells expressing either GFP or ^GFP^ERF3 was induced on KK2 agar for the indicated times, followed by filter trap and western blot analysis (*n* = 3). (**C**) The C-terminal domain is required for ^GFP^ERF3 aggregation during development. Cells expressing either full-length or truncated ^GFP^ERF3 were developed on KK2 agar and then analyzed by filter trap and western blot (*n* = 3). (**D**) ^GFP^ERF3 aggregates at high cell density. Cells serially diluted from high to low starting concentration were incubated in HL5 medium for 24 hours and then subjected to filter trap and western blot analysis (*n* = 3). (**E**) Presence of spent media induces ^GFP^ERF3 aggregation. Spent HL5 medium was collected from wild-type AX4 cells grown to high cell density. *Dictyostelium* cells expressing either GFP or ^GFP^ERF3 were grown in either 100% fresh HL5, 100% spent HL5, 50% spent HL5, or 50% phosphate-buffered medium (PBM)-diluted HL5 and then analyzed by filter trap and western blot (*n* = 3).

In the wild, *Dictyostelium* cells often encounter nutrient-poor conditions. This nutrient stress triggers a developmental cycle that induces *Dictyostelium* cells to transition from unicellular amoeba to multicellular fruiting bodies (Fig. 5A). Because starvation stress can induce aggregation of Sup35 in yeast (12, 39), we wanted to determine if ERF3 aggregation is induced during *Dictyostelium* development. To accomplish this, we induced development of *Dictyostelium* cells overexpressing either GFP or ^GFP^ERF3 on KK2 agar without nutrients and collected lysates at time points along the 24-hour developmental cycle. Strikingly, we observed that ^GFP^ERF3 formed insoluble aggregates within the first 4 hours of development in a manner dependent upon the C-terminal GTPase domain (Fig. 5B and C).

Because induction of *Dictyostelium* development relies on both nutrient deprivation and chemical signaling, we next wanted to determine which of these are required to induce aggregation of ^GFP^ERF3. To accomplish this, we first cultured *Dictyostelium* in rich media at different cell concentrations as higher cell density will result in lower nutrient availability and increased secretion of development-promoting factors following nutrient stress (57–64). Consistent with starvation inducing ^GFP^ERF3 aggregation, we found that ^GFP^ERF3 aggregated in cultures grown at high cell density (Fig. 5D). To determine if exogenous signals are sufficient to drive ^GFP^ERF3 aggregation, we next cultured cells at low density in rich media diluted either with spent media collected from high-density AX4 cultures or with phosphate-buffered medium (PBM). From this, we observed that ^GFP^ERF3 aggregation was induced in the presence of spent media but not in PBM-diluted media (Fig. 5E). Together, these data are consistent with ^GFP^ERF3 aggregation being promoted by chemical signaling during *Dictyostelium* nutrient stress.

## DISCUSSION

While protein aggregation is largely associated with toxicity, it also provides a tunable, epigenetic mechanism that can regulate cellular processes in response to rapid changes in the environment (1–6, 9–12, 65–68). However, the mechanisms that regulate protein aggregation and the cellular pathways that modulate this process are still being uncovered. Here, we have demonstrated stress-induced aggregation of the ERF3 protein in *Dictyostelium discoideum*, an organism highly resistant to protein aggregation (45, 49). Aggregation of ^GFP^ERF3 occurred soon after heat stress and was also induced by several other cellular stressors, indicating that this phenomenon could be part of a rapid and non-specific response to acute stress (Fig. 1). However, unlike its yeast ortholog Sup35, ERF3 did not produce detectable aggregates that propagated to vegetative progeny in a prion-like manner (Fig. 2). Next, because ERF3 aggregates but lacks the N-terminal domain commonly associated with Sup35 aggregation, we wanted to determine if ERF3 was sufficient to aggregate in isolation or if the Q/N-rich proteome of *Dictyostelium* was necessary for its aggregation. To accomplish this, we produced recombinant ERF3 and validated that it aggregated *in vitro*. From this, we observed the formation of ThT-positive assemblies, suggesting that ERF3 may form amyloid *in vitro* similarly to many yeast prions (Fig. 3). To identify the region of ERF3 necessary for ERF3 aggregation, we developed a panel of ERF3 variants consisting of specific ERF3 domains. Using these mutants, we found that ERF3 aggregation was dependent on the C-terminal domain both in *Dictyostelium* cells and *in vitro* (Fig. 4). Because *Dictyostelium* undergo a developmental response under certain types of cellular stress, we also assessed the ability of multicellular development and nutrient stress to induce ERF3 aggregation (Fig. 5). Strikingly, we found that ERF3 formed SDS-insoluble aggregates under conditions that promote development and that this appears to be induced by signals secreted into the media. Together, our findings suggest a role for regulated protein aggregation in response to acute cellular stress in *Dictyostelium* that is both similar to and distinct from *S. cerevisiae*.

Strikingly, we observed that ERF3 aggregated both in stressed *Dictyostelium* cells and *in vitro* despite lacking the Q/N-rich domain necessary for prion formation by *S. cerevisiae* Sup35 (Fig. 1 to 3). Instead, ERF3 aggregation was driven by the ordered C-terminal GTPase domain (Fig. 4). In yeast, Sup35 is capable of entering into heat-induced stress granules independently of its N-terminal prion domain (39, 40). Given that the N-terminal domain is dispensable for ERF3 aggregation in *Dictyostelium* under heat stress (Fig. 4B through D), our findings support these previous reports in suggesting that only the C-terminal domain is required for stress-induced assembly. Recent reports have also found that Sup35 aggregates in yeast cells under starvation conditions in a manner dependent on the C-terminal domain (12, 39). This mirrors our observation that the C-terminal domain of ERF3 is necessary for aggregation during multicellular development in *Dictyostelium* (Fig. 5C), which is induced by nutrient deprivation. Together with these reports, our findings support a conserved role for the C-terminal domain in driving stress-induced ERF3 assembly beyond fungi.

However, it is also widely known that the Sup35 NM domain is capable of forming amyloid and seeding [*PSI+*] prion conversion independently of the C-terminal domain (33, 36, 37). Given that the ERF3 N-terminal domain does not contain the Q/N-rich region present in Sup35, it is perhaps not surprising that we could not detect propagation of ERF3 aggregates to progeny (Fig. 2). An alternate possible explanation for this result is that prion conversion is rare and therefore might be below the level of detection of our assay (29, 33, 69). Taken together with this, our findings support a role for the disordered N and M domains in maintaining ERF3 solubility in *Dictyostelium* rather than helping to drive prion conversion as with *S. cerevisiae* Sup35. It is possible that addition of the Q/N-rich prion domain from yeast Sup35 to *Dictyostelium* ERF3 would result in a gain of prion formation capability by ERF3. However, previous work has found that *Dictyostelium* is resistant to aggregation of the Sup35 NM domains under normal conditions (45). This differs greatly from yeast and other organisms in which these domains are highly aggregation prone, indicating that the effect of this domain on prion formation may also differ in *Dictyostelium* (45). Additionally, given that we did not observe formation of ^GFP^ERF3 foci by microscopy, it is possible that the aggregates formed by this protein are large enough to be detected biochemically but not large enough to overcome size thresholds for prion seeding (70). It is also worth noting that the context of the unusually Q/N-rich proteome of *Dictyostelium* varies greatly from that of *S. cerevisiae* (45, 46, 49). Thus, it is likely that there would be unique proteostatic processes regulating *bona fide* prion formation in *Dictyostelium*, which could be a promising avenue for further study.

Our observation of ERF3 aggregating independently of a Q/N-rich region raises the question of whether stress-responsive aggregation of ERF3 orthologs occurs more broadly across eukaryotes. While there tends to be high variability in the N and M domain sequences, there is stronger sequence conservation of the C-terminal GTPase domain across evolutionarily diverged eukaryotes (71, 72). Besides sequence conservation, there are also distinct features of these orthologous proteins that are shared by distantly related organisms. For instance, the human eukaryotic release factor encoded by the gene GSPT1 resembles *Dictyostelium* ERF3 and *S. cerevisiae* Sup35 in that it is similarly disordered in the N-terminus and contains a negatively charged region upstream of the C-terminal GTPase (Fig. S1A and B). Alignment of predicted protein structures also suggests that the C-terminal region of GSPT1 closely resembles those of *Dictyostelium* ERF3 and *S. cerevisiae* Sup35 (Fig. S1C). However, aggregation of GSPT1 in response to cellular stress has not been examined despite the many impacts this phenomenon could have on cellular function. In the future, it will be important to consider expanding investigation of eukaryotic release factor assembly beyond fungi and *Dictyostelium*.

The assemblies formed by recombinant ERF3 *in vitro* were ThT positive (Fig. 3E), indicating that the protein may be forming amyloid (73). However, the branching ERF3 assemblies observed by TEM lack the characteristic order of amyloid fibrils, suggesting that the protein may instead be forming amorphous aggregates (Fig. 3F). Therefore, it is possible that aggregation of ERF3 may manifest similarly to bacterial inclusion bodies, which have characteristics of both amyloid and amorphous aggregates (74–77). As with bacterial inclusion bodies, this raises questions on how aggregation and amyloid-like characteristics impact the cellular toxicity and the function of these proteins.

While amyloid is often associated with deleterious events, there are also functional amyloids formed by cells that play important roles in cellular processes. These include structural roles, cellular signaling, and other important functions within the cell (78–82). Our findings raise the question of whether ERF3 forms functional amyloid within *Dictyostelium* cells under stress. In this case, there is also the question of whether formation of functional amyloids could occur on a greater scale within the highly Q/N-rich and prion-like proteome of *Dictyostelium*. In the future, further work into understanding stress and developmentally induced protein aggregation in *Dictyostelium* is warranted and may reveal novel mechanisms that regulate protein solubility.

Adaptability is especially important for soil-dwelling organisms such as *Dictyostelium* that encounter highly dynamic environmental conditions as a result of their habitat. Our observation of stress-induced ERF3 aggregation in *Dictyostelium* mirrors findings made in yeast on the potential fitness benefits of protein aggregation. Thus, the potential cellular consequences of ERF3 aggregation in *Dictyostelium* stress response may resemble those of Sup35 assembly in yeast. For instance, Sup35 and associated translational machinery can assemble into reversible stress granules following heat stress, resulting in transient suppression of translational activity (39, 40, 83). Aggregation of Sup35 in yeast cells under stress also results in a faster recovery of translational activity following removal of the stress (12). In addition, translational readthrough that occurs when Sup35 is in its prion form can result in distinct protein products (84–88). It is possible that aggregation of ERF3 in *Dictyostelium* cells under stress could result in similar translational readthrough. Moving forward, it will be important to understand how ERF3 aggregation alters protein function in *Dictyostelium*.

Alternatively, other unique benefits to the cells may result from the induction of protein aggregation in *Dictyostelium*. For example, nutrient stress induces a shift to a more gel-like cytoplasm in both yeast and *Dictyostelium* cells, and this phenomenon aids in recovery from dormancy (9). Given that ERF3 assembles in response to multicellular development (Fig. 5), the induction of protein aggregation may also have a functional role in regulating the biology of *Dictyostelium* cells during development, differentiation, and/or entry into dormancy. Likewise, given that *Dictyostelium* live in complex microbial ecosystems and prey on bacteria, there may be cross-kingdom interactions influencing the behavior of prion-like proteins within *Dictyostelium* as occurs in yeast (89–93). In the future, further investigation is needed to elucidate the pathways regulating the induction, clearance, and functional consequences of protein aggregation in *Dictyostelium* biology.

## MATERIALS AND METHODS

### Protein sequence analyses

*Dictyostelium discoideum* ERF3, *S. cerevisiae* Sup35, and human GSPT1 protein sequences were obtained from UniProt (Q7YZN9, P05453, and P15170, respectively). Predicted structures were obtained from AlphaFold (94, 95) and aligned in PyMOL v2 (Schrödinger, LLC.). Disorder prediction was conducted using IUPRED3 (96), and protein charge analysis was performed using CIDER (97). Protein sequence alignment was performed using ClustalOmega (98) and visualized in Jalview (99).

### *Dictyostelium* cell culture

*Dictyostelium discoideum* axenic strain AX4 cells were cultured in HL5 medium [per 1 L: 17.8 g proteose peptone (Millipore #107229), 7.2 g yeast extract (BD #212720), 0.54 g Na_2_HPO_4_ (J.T. Baker cat. #3824-01), 0.4 g KH_2_PO_4_ (Sigma #P9791), pH 6.5] supplemented with 130 µL B12/folic acid mix [per 100 mL: 5 mg B12 (Sigma-Aldrich #V2876) and 200 mg folic acid (Sigma-Aldrich #F7876)], 20 mL of 50% glucose (Sigma-Aldrich #G8270), 300 µg/mL streptomycin (GoldBio #S-150-100), and 100 µg/mL carbenicillin (GoldBio #C-103-50). Cells were split routinely to prevent cultures from reaching densities higher than 4 × 10^6^ cells/mL. Overexpression constructs were transformed into AX4 cells by electroporation following established protocols (100). For selection of electroporated cells, HL5 was additionally supplemented with 100 µg/mL G418 (GoldBio #G-418-25).

### Plasmids

To construct the ^GFP^ERF3 plasmid for *Dictyostelium* expression, the ERF3 gene was PCR amplified from AX4 cDNA and inserted into the pTXGFP vector using BamHI-HF (NEB #R3136) and XhoI (NEB #R0146). For expression of the GFP-tagged ERF3 truncations, plasmids were constructed using the GoldenBraid system (101). Prior to GoldenBraid assembly, the ERF3 gene was PCR amplified and then TOPO TA cloned into the pCR4 vector (Invitrogen #K457502). The gene was then mutagenized using the Quikchange II Kit (Agilent #200522) to remove a BsmBI enzyme site by introducing a silent c1305t point mutation. Truncated forms of ERF3 were PCR amplified from this BsmBI-null template and then domesticated into the pUPD2 vector using BsmBI-v2 (NEB #R0739). The genes were then assembled into an expression cassette in the A1 vector with a coaA promotor, C-terminal sfGFP, and act8t terminator using BsaI-HFv2 (NEB #R3733). For construction of the final expression vectors, these cassettes were then assembled with the DdExChr extrachromosomal maintenance marker in the O2N vector. All GoldenBraid parts and expression vectors were acquired from the Dictyostelium Stock Center (102). Htt^ex1^ was expressed recombinantly in BL21 cells using the pET32a-Htt^ex1Q46^ plasmid [Addgene #11515 (103)]. For bacterial expression of ERF3, the gene was PCR amplified and then inserted into the pET32a-Htt^ex1Q46^ plasmid using NcoI-HF (NEB #R3193) and SalI-HF (NEB #R3138) to remove the Htt fragment while retaining the Trx tag and 6xHis tag. The truncated ERF3 plasmids for bacterial expression were assembled using the same method.

### *Dictyostelium* stress experiments

For heat stress experiments, *Dictyostelium* cells were plated in 10-cm petri dishes at 1 × 10^6^ cells/mL and then incubated in a water bath at the indicated temperatures for 2 hours. Cells were then resuspended and collected by centrifugation at 500 × *g* for 3 minutes. Media were discarded, and then, cells were washed once with cold 1× PBS before being lysed in RIPA buffer [50 mM Tris pH 8.0, 150 mM NaCl, 0.1% SDS, 0.5% sodium deoxycholate, 1% IGEPAL CA-630, protease inhibitor cocktail (1:100, Sigma #P8340), and PMSF (1:1,000, RPI #P20270)]. For experiments to assess the persistence of aggregates following heat stress, cells were incubated at either 22°C, 27°C (*n* = 2), or 30°C (*n* = 2) for 2 hours in 10-cm dishes at 1 × 10^6^ cells/mL. These cells were then collected, diluted to 2.5 × 10^5^ cells/mL in HL5, and grown in shaking flasks at 22°C. Samples were collected, and cultures were diluted to 2.5 × 10^5^ cells/mL to prevent overgrowth every 24 hours. For pH stress experiments, cells were incubated at 22°C for 24 hours in HL5 at the indicated pH and then collected by centrifugation, washed once with PBS, and lysed in RIPA buffer. HL5 pH was adjusted using HCl. For other cellular stressors, AX4 cells were subjected to a range of stressor dosages for 18 hours followed by measurement of optical density at 600nm to determine the half-maximal inhibitory concentration (IC50) of growth for each stressor. *Dictyostelium* cells expressing either GFP or ^GFP^ERF3 were then incubated for 18 hours at 22°C in HL5 containing each of the following stressors at the indicated IC50 concentration: 200 µM CdCl_2_, 2% ethanol, 100 mM MgCl_2_, or 0.15 M NaCl. Cells were then collected by centrifugation, washed once with PBS, and lysed in RIPA buffer.

### *Dictyostelium* development

Multicellular development of *Dictyostelium* cells was induced following the standard protocols from Dictybase (104). Cells were washed three times with cold development buffer [5 mM Na_2_HPO_4_, 5 mM KH_2_PO_4_, 1 mM CaCl_2_, and 2 mM MgCl_2_] prior to plating on KK2 agar [per 1 L: 2.2 g KH_2_PO_4_, 0.7 g K_2_HPO_4_, and 15 g agar]. Plates were incubated at 22°C for the specified length of time before cells were collected and washed in PBS, followed by lysis in RIPA buffer.

### SDS-PAGE, SDD-AGE, and filter trap assays

Total protein concentrations in lysates were determined by BCA (Thermo Scientific #23225). For all assays, 20 µg of lysate protein per sample was used unless otherwise indicated. For SDS-PAGE, samples were prepared for loading by adding an appropriate volume of 4× Laemmli buffer to lysate protein. Samples and protein size ladder (Thermo Scientific #26619) were run on 4%–20% gradient SDS-polyacrylamide gels at 185 V and then transferred to PVDF membrane (Bio-Rad #1620177) overnight at 30 V for analysis by western blot. SDD-AGE was performed following established protocols (105). Samples were prepared for loading by adding an appropriate volume of SDD-AGE sample buffer to lysate protein. Gels were run at 75 V for 5 hours at 4°C followed by capillary transfer to a nitrocellulose membrane (Bio-Rad #1620115) for analysis by western blot. For filter trap assay, lysate protein was diluted to 90 µL with RIPA buffer. Ten microliters of 10% SDS was then added to each sample, followed by 900 µL of 1% SDS in PBS for a final volume of 1 mL. Samples were then applied to a 0.2-µm cellulose acetate membrane (Sterlitech #CA023001) on a DHM-48 dot blot manifold by vacuum. Samples were each washed with 1 mL of 1% SDS in PBS; then, membranes were assessed by western blot.

### Western blotting

Membranes were blocked for 30 minutes using 5% milk in TBS buffer with 0.1% tween (TBST) prior to incubation with primary antibody for 3 hours at room temperature or overnight at 4°C. Primary antibodies were prepared in 5% milk in TBST at the indicated concentrations. Primary antibodies used were α-GFP (1:1,000, Invitrogen #A-11122), α-6xHis (1:1,000, Invitrogen #MA1-21315), and α-beta actin (1:100, DSHB #224-236-1, deposited to the DSHB by G. Gerisch).

After incubation with primary antibody, the membranes were washed three times for 10 minutes with TBST followed by incubation with secondary antibody for 1 hour at room temperature. Membranes were washed three times for 10 minutes with TBST then incubated briefly in 10 mL of enhanced chemiluminescence buffer [50 mM Na_2_HPO_4_, 50 mM Na_2_CO_3_, 10 mM NaBO_3_·4H_2_O, 250 mM luminol, 90 mM coumaric acid] activated with 8 µL of 30% hydrogen peroxide (Macron #5240-02). Membranes were imaged using a Bio-Rad ChemiDoc MP system.

### Confocal microscopy

*Dictyostelium* cells were transferred to live-cell imaging chambers at 1 × 10^6^ cells/mL and allowed time to adhere before HL5 medium was replaced with low fluorescence medium (ForMedium #LF0501). Images were captured before and after a 2-hour heat stress at 30°C using a Zeiss 880 inverted confocal microscope with Airyscan detection.

### Recombinant protein purification

Recombinant Htt^ex1Q46^ was purified from BL21 cells as previously described (48, 106). Recombinant ERF3 was purified from Rosetta cells grown to an optical density of 0.6 at 37°C and then induced overnight at 16°C with 1 mM IPTG (GoldBio #I2481). Cells were collected by centrifugation at 4,000 × *g* for 10 minutes and then resuspended in 25 mL of Buffer A (50 mM Tris pH 7.5, 1 M NaCl, and 0.5% Triton X-100) per liter of cells. Lysozyme was added to improve lysis before tumbling cells for 30 minutes at 4°C. Lysates were sonicated and then centrifuged at 20,000 × *g* for 30 minutes. Supernatant was added to 500 µL nickel HTC agarose beads (GoldBio #R-202-500) per 1 L cells pre-washed three times with Buffer A. Beads were tumbled for 1–3 hours at 4°C and then washed three times with Buffer A, three times with Buffer B (50 mM Tris pH 7.5, 1 M NaCl, and 20 mM imidazole), and three times with ThT assay buffer (20 mM Tris-HCl pH 8.0, 50 mM NaCl, and 2 mM CaCl_2_) containing 20 mM imidazole. Protein was eluted from the beads by tumbling 15 minutes at 4°C in an equal volume of ThT assay buffer containing 300 mM imidazole. Eluates were centrifuged at 14,000 rpm for 15 minutes to remove insoluble content. Protein concentration was quantified by NanoDrop.

### Thioflavin-T aggregation assays

ThT aggregation assays were conducted as previously described (48, 106). Reactions were prepared on ice in ThT assay buffer. ThT (Sigma-Aldrich #T3516) was added to a concentration of 10 µM, and the specified recombinant protein was added to a concentration of 15 µM. Enterokinase (NEB #P8070L) was added at 1.6 units per 50 µL reaction to initiate aggregation. Each reaction was then added to a black, clear-bottomed 384-well plate in three 50-µL aliquots. Reactions proceeded for 16–18 hours at 37°C, and fluorescence was measured every 30 minutes with excitation at 440 nm and emission at 480 nm using a Tecan Spark plate reader. Plates were briefly agitated prior to each measurement.

### Transmission electron microscopy

Recombinant ERF3 at 15 µM in ThT assay buffer was mixed with enterokinase at 1.6 units/50 µL and incubated overnight at 37°C to allow aggregation to occur. These samples were then diluted to 0.5–2 µM in ThT assay buffer to prevent overloading on the TEM grids. Formvar/carbon TEM grids (Electron Microscopy Sciences #FCF300-Cu-50) were discharged using a PELCO easiGlow system. Ten microliters of the aggregated ERF3 sample was applied to the grid for 30 seconds before being gently wicked off. Grids were then negative stained for 30 seconds using a 2% aqueous uranyl acetate solution (provided by Duke Shared Materials Instrumentation Facility). Excess stain was gently wicked off, and grids were allowed to air dry. Grids were imaged on a FEI Tecnai G² Twin microscope using the Gatan DigitalMicrograph software.

## Data Availability

All plasmids generated in this work are available from the Dictyostelium Stock Center. Raw data are available upon request.

## SUPPLEMENTAL MATERIAL

Supplemental material spectrum.01607-24-s0001.pdf Fig. S1 and S2.

ASM does not own the copyrights to Supplemental Material that may be linked to, or accessed through, an article. The authors have granted ASM a non-exclusive, world-wide license to publish the Supplemental Material files. Please contact the corresponding author directly for reuse.
