# Supplementary material for: Acute stress and multicellular development alter the solubility of the Dictyostelium Sup35 ortholog ERF3
Source: Microbiol Spectr. 2024 Sep 30;12(11):e01607-24. doi: 10.1128/spectrum.01607-24 (PMC11537047; doi:10.1128/spectrum.01607-24)
Supplement: Supplemental material — Fig. S1 and S2. [file spectrum.01607-24-s0001.pdf]

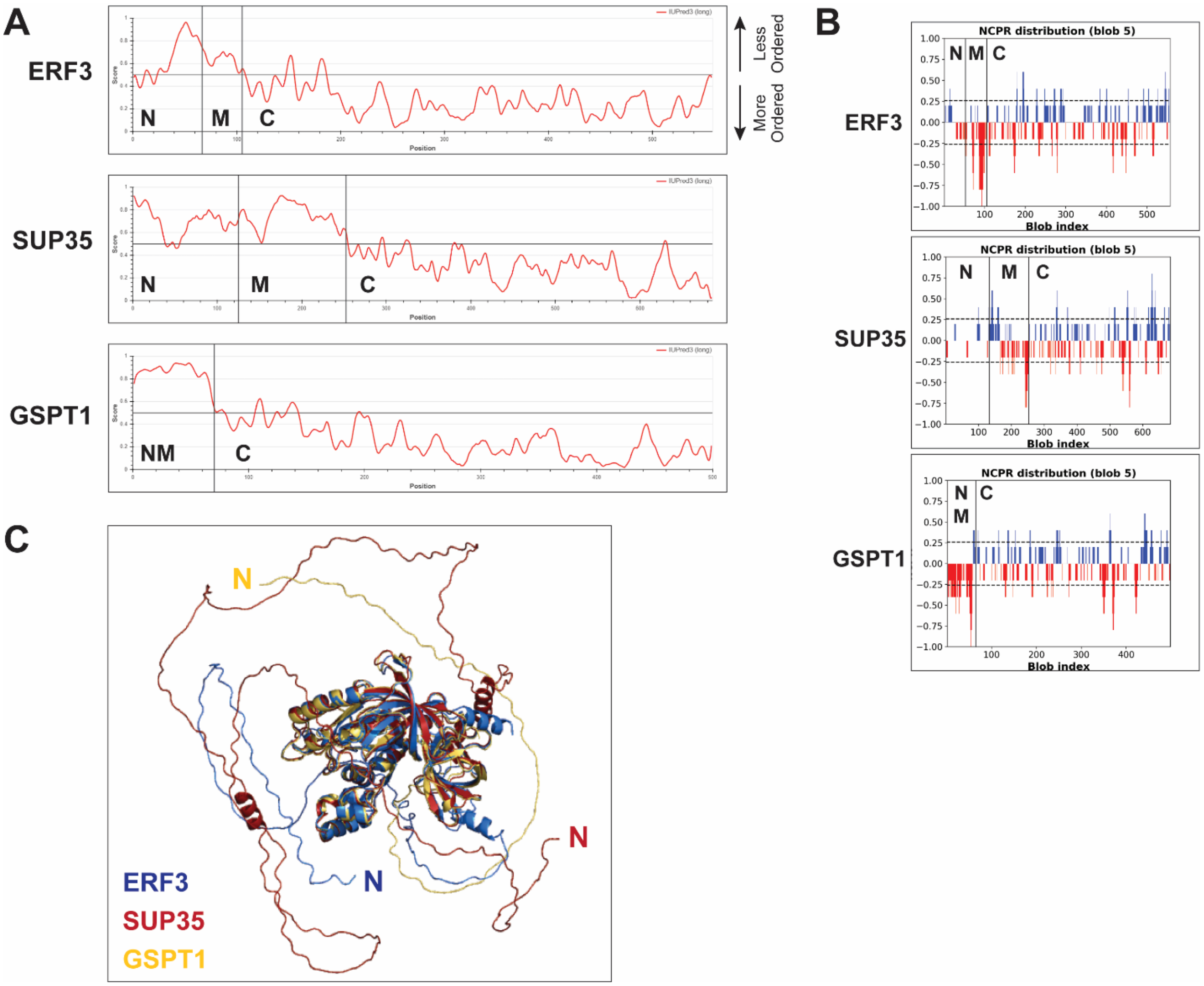

SUPPLEMENTARY FIG 1 Certain protein features are shared across divergent eukaryotic release factors. (**A**) *Dictyostelium discoideum* ERF3, *Saccharomyces cerevisiae* Sup35, and human GSPT1 are similarly disordered in the N-terminus. Protein disorder prediction was performed using IUPRED3. Scores above the indicated threshold represent predicted disordered regions. (**B**) ERF3, Sup35, and GSPT1 contain negatively charged regions. Distribution of net charge per residue (NCPR) was analyzed using CIDER. Red peaks indicate negatively charged clusters and blue peaks indicate positively charged clusters. (**C**) Human GSPT1 is structurally similar to *Dictyostelium* ERF3 and *S. cerevisiae* Sup35 in the C-terminal region. Protein structures predicted by AlphaFold and aligned in PyMOL.

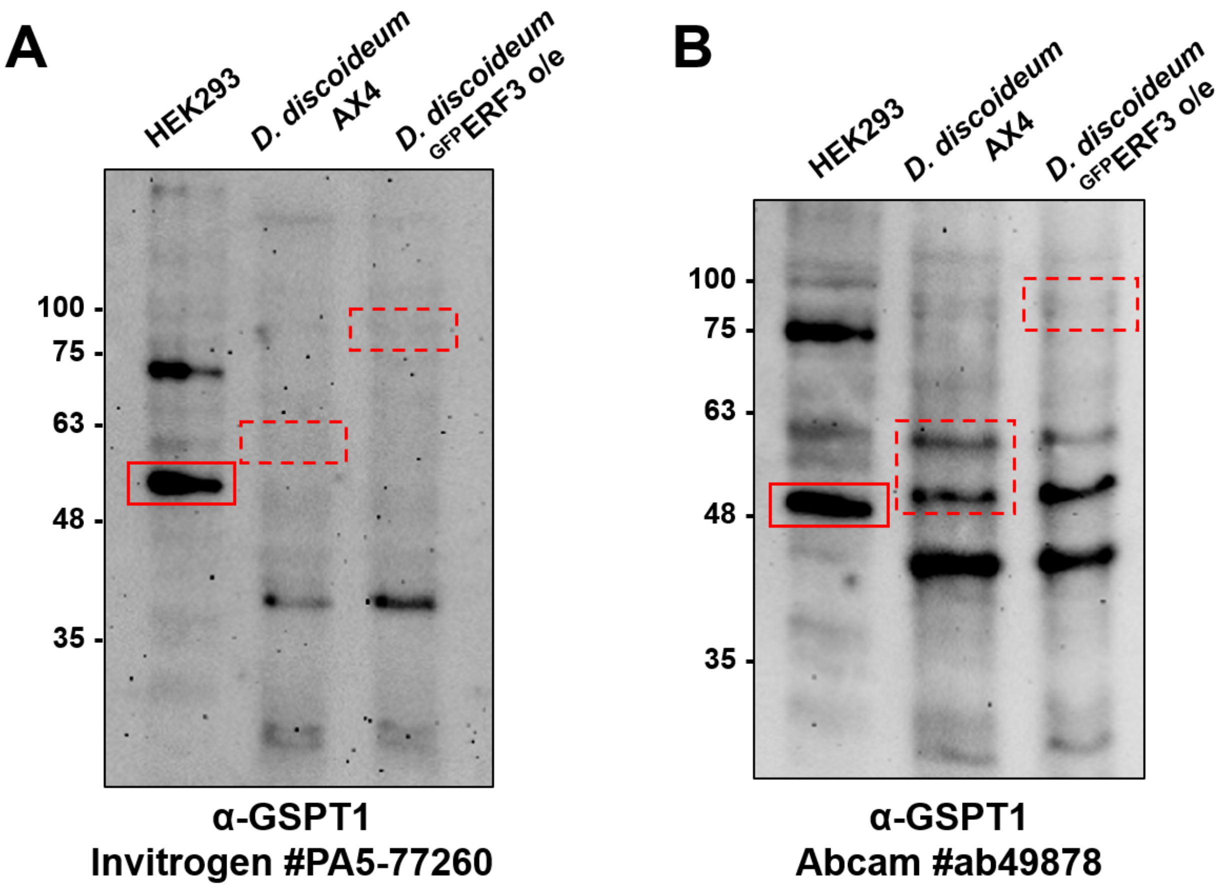

SUPPLEMENTARY FIG 2 Antibodies raised against partially homologous regions of human GSPT1 do not detect *Dictyostelium* ERF3. Samples are lysates from human-derived HEK293 cell line (10µg, expected size 55kDa), *Dictyostelium* AX4 cells (40µg, expected size 60kDa), and *Dictyostelium* cells overexpressing ^{GFP}ERF3 (40µg, expected size 88kDa). Invitrogen #PA5-77260 (A) was successful in detecting human GSPT1 but did not produce bands of the expected size for *Dictyostelium* ERF3. While Abcam #ab49878 (B) did produce bands in the expected size range for endogenous ERF3, there was not a band in the expected size range for ^{GFP}ERF3, suggesting that the bands between 48 – 63 kDa were likely non-specific.
